# Association of α-klotho levels with serum copper and cadmium levels in schizophrenia

**DOI:** 10.1192/j.eurpsy.2023.589

**Published:** 2023-07-19

**Authors:** D. Yadav, A. Birdi, N. Nebhinani, K. Kumar, S. Tomo

**Affiliations:** ^1^Biochemistry; ^2^Psychiatry, All India Institute of Medical Sciences, Jodhpur, India

## Abstract

**Introduction:**

Schizophrenia is associated with harmful health effects such as oxidative stress from heavy metal exposure. We considered the relationship between genes and heavy metals in association with oxidative stress and then investigated the association between serum α- klotho and copper and cadmium exposure among schizophrenia patients in western India.

**Objectives:**

To investigated the association between serum α- klotho and copper and cadmium exposure among schizophrenia patients in western India.

**Methods:**

100 individuals participated out of which 50 were diagnosed with schizophrenia, severity was assessed by using PANSS score and 50 were taken as controls using General health questionnaire. Serum Klotho levels were estimated using ELISA. Serum Cadmium (Cd) and Serum Copper (Cu) was estimated using Atomic Absorption Spectrophotometry.

**Results:**

The mean ± SD levels of Serum Cd, Serum Cu and serum Klotho were 1.05 ± 0.55 μg/dl, 135.5 ± 51.25 μg/ml and 62.9 ± 35.1 ng/ml respectively in the patients and 0.23 ± 0.17 μg/dl, 147.9 ± 25.42 μg/ml and 78.6 ± 34.6 ng/ml respectively in controls. The differences in Serum Cd, Serum Cu levels and Klotho levels among the study group were highly significant (p <0.05).

**Conclusions:**

Both Cu and Cd levels were significantly raised in schizophrenic patients compared with controls. Serum klotho levels showed a statistically significant decreasing trend with increasing cadmium levels. These results suggest that cadmium levels may be associated with the serum klotho levels which may be associated with decreased cognition in schizophrenia.

**Disclosure of Interest:**

None Declared

